# Evaluating the value of individualized 3D printed models for examination, diagnosis and treatment planning of cervical cancer

**DOI:** 10.1186/s41205-024-00229-8

**Published:** 2024-07-27

**Authors:** Anne Cathrine Scherer-Quenzer, Inga Beyers, Adam Kalisz, Stephanie Tina Sauer, Marcus Zimmermann, Achim Wöckel, Bülent Polat, Tanja Schlaiss, Selina Schelbert, Matthias Kiesel

**Affiliations:** 1 Department of Obstetrics and Gynecology, University Hospital of Wuerzburg, Josef-Schneider-Strasse 4, Würzburg, 97080 Germany; 2 Institute of Electric Power Systems (IfES), Leibniz University Hannover, Appelstraße 9A, Hannover, 30167 Germany; 3 Department of Electrical, Electronic and Communication Engineering, Information Technology (LIKE), Friedrich-Alexander-University Erlangen-Nuernberg, Am Wolfsmantel 33, Erlangen, Germany; 4 Department of Diagnostic and Interventional Radiology, University Hospital Wuerzburg, Oberduerrbacher Straße 6, Würzburg, 97080 Germany; 5 Department of Radiation Oncology, University Hospital Wuerzburg, Josef-Schneider-Str. 11, Würzburg, 97080 Germany; 6 Institute of Pathology, University of Wuerzburg, Josef-Schneider-Straße 2, Würzburg, 97080 Germany

**Keywords:** 3D printing, Visualization, Cervical cancer, Treatment planning, Interprofessional communication, Staging, Translational oncology, MRI

## Abstract

**Background:**

3D printing holds great potential of improving examination, diagnosis and treatment planning as well as interprofessional communication in the field of gynecological oncology. In the current manuscript we evaluated five individualized, patient-specific models of cervical cancer FIGO Stage I-III, created with 3D printing, concerning their value for translational oncology.

**Methods:**

Magnetic resonance imaging (MRI) of the pelvis was performed on a 3.0 Tesla MRI, including a T2-weighted isotropic 3D sequence. The MRI images were segmented and transferred to virtual 3D models via a custom-built 3D-model generation pipeline and printed by material extrusion. The 3D models were evaluated by all medical specialties involved in patient care of cervical cancer, namely surgeons, radiologists, pathologists and radiation oncologists. Information was obtained from evaluated profession-specific questionnaires which were filled out after inspecting all five models. The questionnaires included multiple-select questions, questions based on Likert scales (1 = „strongly disagree “ or „not at all useful “ up to 5 = „strongly agree “ or „extremely useful “) and dichotomous questions (“Yes” or “No”).

**Results:**

Surgeons rated the models as useful during surgery (4.0 out of 5) and for patient communication (4.7 out of 5). Furthermore, they believed that the models had the potential to revise the patients’ treatment plan (3.7 out of 5). Pathologists evaluated with mean ratings of 3.0 out of 5 for the usefulness of the models in diagnostic reporting and macroscopic evaluation. Radiologist acknowledged the possibility of providing additional information compared to imaging alone (3.7 out of 5). Radiation oncologists strongly supported the concept by rating the models highly for understanding patient-specific pathological characteristics (4.3 out of 5), assisting interprofessional communication (mean 4.3 out of 5) and communication with patients (4.7 out of 5). They also found the models useful for improving radiotherapy treatment planning (4.3 out of 5).

**Conclusion:**

The study revealed that the 3D printed models were generally well-received by all medical disciplines, with radiation oncologists showing particularly strong support. Addressing the concerns and tailoring the use of 3D models to the specific needs of each medical speciality will be essential for realizing their full potential in clinical practice.

**Supplementary Information:**

The online version contains supplementary material available at 10.1186/s41205-024-00229-8.

## Background

Cervical cancer represents the fourth leading cancer-entity among women worldwide, with 604,127 new cases in 2020 [1]. Treatment options include surgery, chemoradiotherapy, chemotherapy and radiotherapy (Cervical Cancer: ESMO Clinical Practice Guidelines). Treatment decision mainly depends on tumor size and tumor spread, but also age, general state of health and family planning are considered. Selection of the appropriate therapy is made by a multidisciplinary team of surgeons, radiologists and radiation oncologists and requires an accurate understanding of the patient’s uterine anatomy, tumor location and disease progression.

So far, gynecologic pelvic examination and medical imaging are the only means to provide the above-mentioned information. Conventional diagnostic methods rely on 2D imaging modalities, presenting the complexity of cervical tumors only to a limited extent. Moreover, the interpretation of a large number of 2D slices is dependent on the experience of the examining physician and is time-consuming [2]. In contrast, the utilization of 3D printed models, based on MRI segmented scans, is expected to provide high-resolution anatomical insights allowing a more comprehensive visualization of the patients’ individual cervical cancer disease [3]. Until now, there is only limited data on the use of 3D printed models in daily clinical practice, mainly in the fields of dentistry [4, 5]. Yet, there are studies showing that 3D printed teaching models of cervical cancer can be helpful for postgraduate gynecological training [2, 6–8]. The present work aims to evaluate the effectiveness and value of 3D printed models in improving the understanding of cervical cancer progression, aiding in diagnosis and facilitating more tailored and efficient treatment planning strategies. It was also investigated whether the 3D visualization could improve the interprofessional communication between surgeons, radiologists, pathologists and radiation oncologists. Moreover, the study evaluated whether these models could serve as a tool for patient education enabling more informed discussions and shared decision-making.

## Methods

### Subjects and acquisition conditions

This study was registered with the Institutional Review Board (Ref. No. 20200910 02). Five patients (mean age 38 years; age range 27–47 years) with histologically confirmed cervical cancer were included in the study. MRI of the pelvis as indicated by the national guidelines for locoregional staging [9] was performed on a 3.0 Tesla MRI System (Magnetom Skyra, Siemens Healthcare, Erlangen, Germany) in supine patient position. Following the recommendations of the European Society of Urogenital Radiology [10] a multiparametric approach, including but not limited to two-dimensional T2-weighted sequences, diffusions-weighted imaging and contrast-enhanced imaging (Gadovist, Bayer Vital GmbH, Germany; 0.1 mmol/kg body weight) was used. Additionally, a coronar T2-weighted isotropic 3D fast turbo spin echo sequence was acquired (in plane resolution 0.9 mm × 0.9 mm, slice thickness 1 mm, field-of-view 300 mm × 300 mm) and reconstructed in transversal and sagittal orientation. This sequence was exported anonymized and used for segmentation.

### Segmentation

**Fig. 1:**
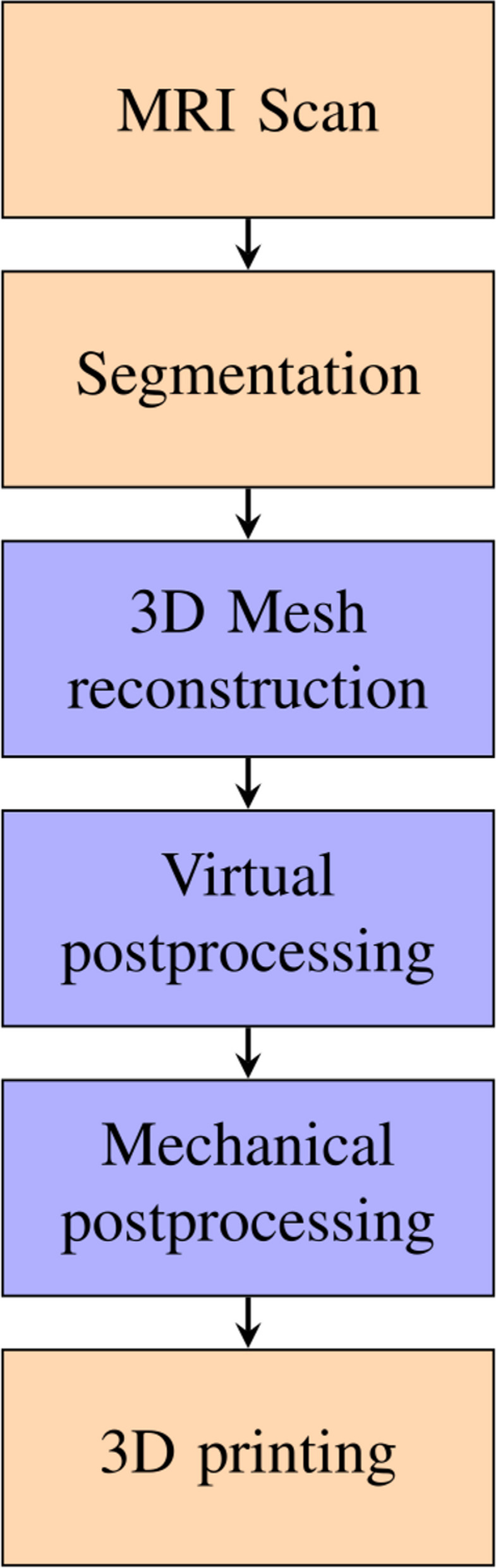
Our proposed pipeline of creating individualized 3D printed models. Orange: manual steps. Blue: Automated steps

The procedure from MRI scan to final print is depicted in Fig. 1. Based on the MRI scans, one board-certified radiologist with more than 5 years of experience in gynecologic pelvic imaging segmented uterus, bladder, rectum and cervical cancer. The segmented data was then processed by our own software (written in *Python*, Van Rossum, G., & Drake Jr, F. L. (1995), Python reference manual, Centrum voor Wiskunde en Informatica Amsterdam). Our software read in the dataset using the *PyDicom* library, extracted the individual slices and passed them on to a Marching Cubes algorithm. This algorithm was provided by the *SKImage.measure* library which generated a list of vertices and facets (i.e. triangles). Due to the underlying voxel grid used in the marching cubes algorithm, the surface is usually not continuously smooth. Therefore we first processed the mesh in *Blender* (Community, B. O. 2018. Blender—a 3D modelling and rendering package. Stichting Blender Foundation, Amsterdam) by decimating the surface by a constant factor. We then applied Laplacian smoothing to the surface. The resulting virtual model was then exported from *Blender* as a.stl file for printing.

### 3D Printing

The resulting virtual 3D models, created from the segmented MRI scans, were subsequently 3D printed to create haptic and real-life representations. The overarching aim in the 3D printing process was to give the final products a desktop-model-like character. Therefore, a weighted stand was designed to mount the organs with the correct orientation and its base inscribed with the respective Study ID. The organs were printed at life-size scale.

One challenge which needed to be resolved was an organ overlap in the 3D-model resulting from imperfect scanning and extraction. This organ overlap was removed with Boolean operations, following a set logic sequence. The carcinoma was kept complete and no modifications to its shape were allowed, so as not to compromise operation planning. In case of an overlap with the uterus, the overlapping volume was removed from the uterus. In case of overlaps between uterus and bladder or uterus and rectum, the overlap volume was attributed to the uterus and cut out of the respective other organ.

**Fig. 2:**
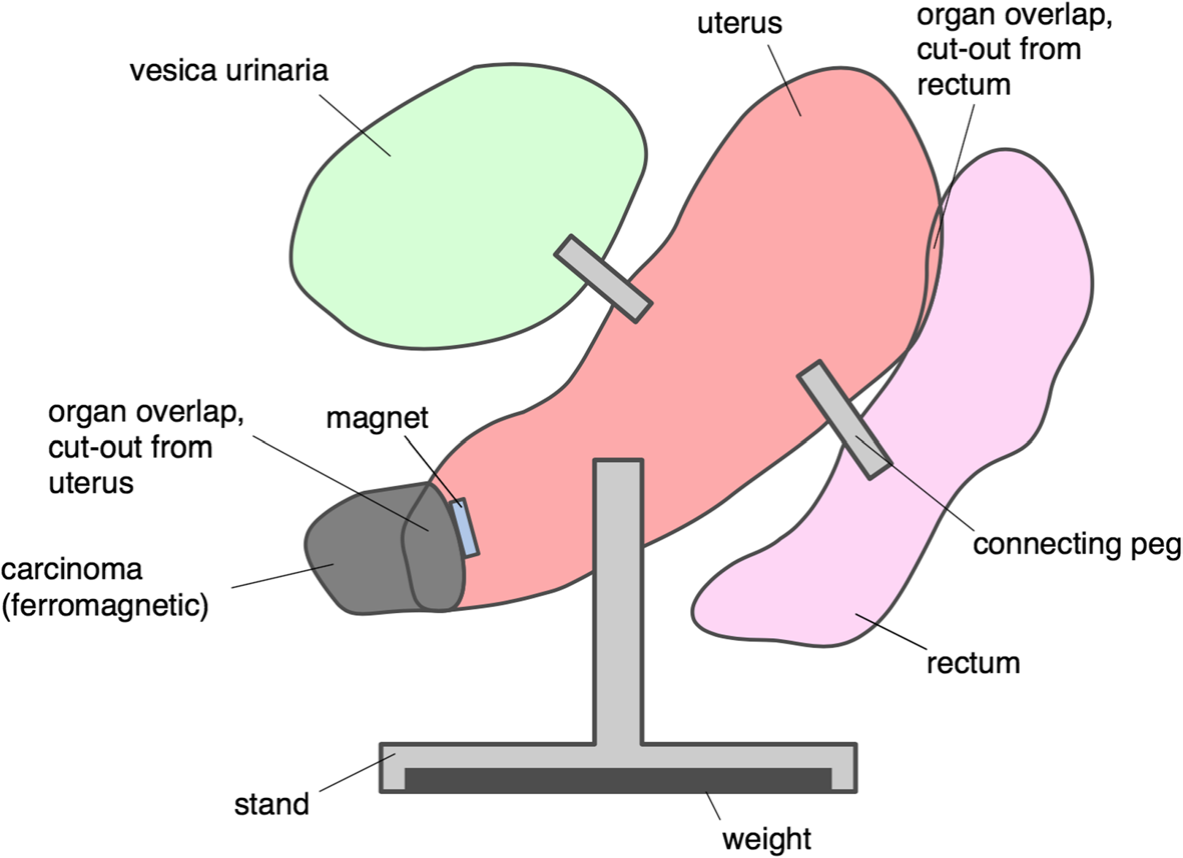
Depiction of the anatomical structures which were segmented from MRI scans, 3D printed and attached by connecting pegs and a magnetic pin. Green: Bladder. Red: Uterus. Grey: Cervical cancer. Pink: Rectum

The different model elements are mechanically fastened to each other but can be taken apart as well. The model base inserts into the lowest-lying organ (uterus or rectum) via a press fit. The rectum and bladder connect to the uterus via custom round pegs. The connecting pegs have a notch, so that the respective organs can only fit in one correct position, thereby ensuring an unambiguous orientation. Because one goal was to leave the carcinoma shape unaltered, this implies that no cut-outs for mechanical fastening are allowed in the carcinoma, presenting a challenge to the model design. This was solved by printing the entire carcinoma from ferromagnetic material and inserting magnets into the uterus at the uterus-carcinoma area of contact. An overview of the mechanical model design is given in Fig. 2.

**Fig. 3:**
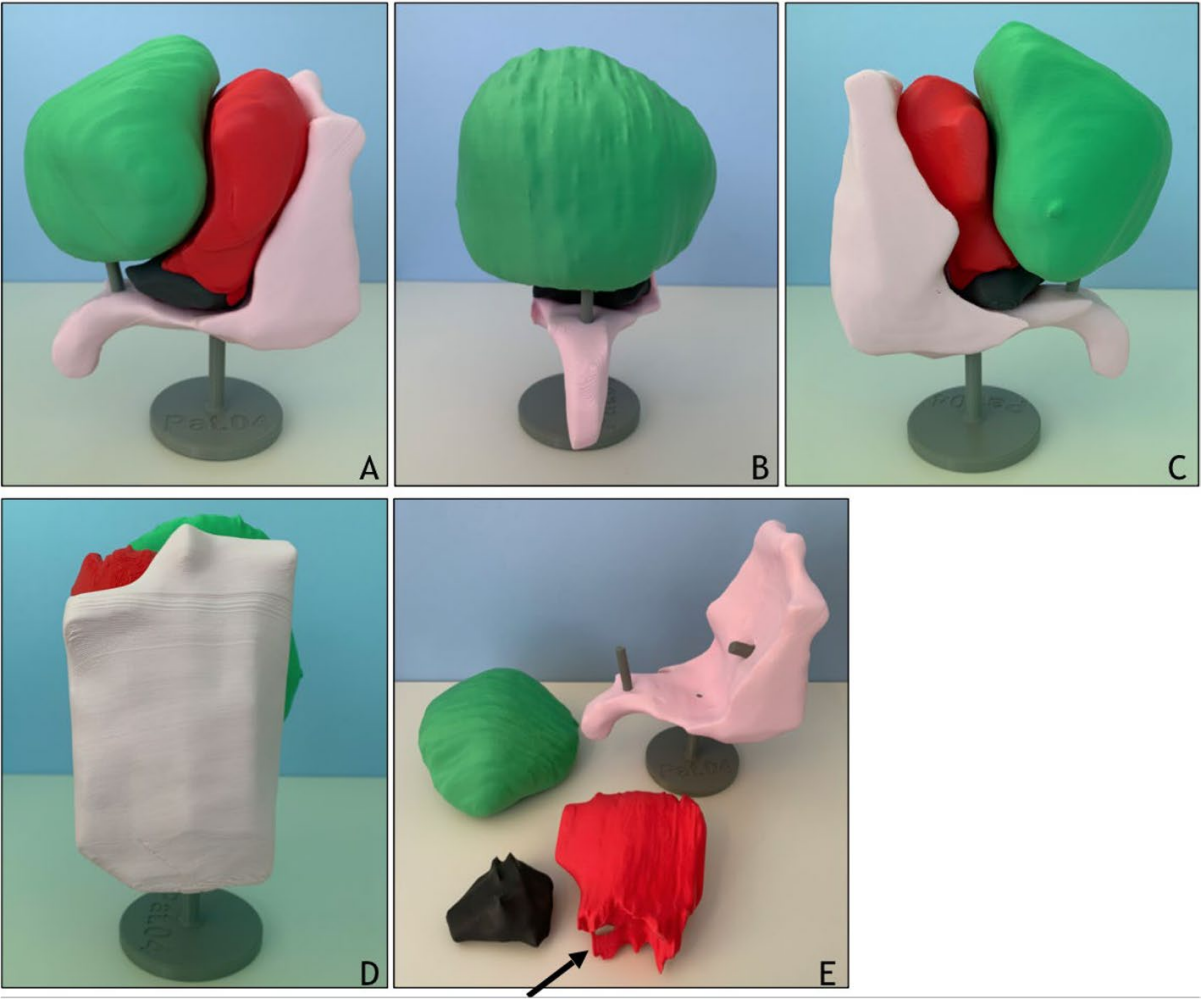
Depiction of one 3D printed model. Picture taken from **A** right side, **B** front, **C** left side, **D** backside. **E** Shows all single structures. Green: Bladder. Red: Uterus. White: Rectum. Black: Cervical cancer. Arrow shows magnetic pin

The model elements were 3D printed via fused deposition modeling (FDM) on Prusa MK3s and Prusa Mini machines (Prusa, Praha, Czech Republic). The same filament was used to color-code the different elements across all models. This helps to quickly identify the organs, as the shapes and orientation vary considerably between patients. The filaments used were Polymaker PolyTerra PLA, type “Lava Red” for the uterus; Polymaker PolyTerra PLA, type “Sakura Pink” for the recta; Polymaker PolyTerra PLA, type “Forrest Green” for the bladder (Polymaker, Changshu, China); Filamentworld PLA “Grey” for the stand and connecting pegs (Filamentworld, Neu-Ulm, Germany); Proto-Pasta, type “Magnetic Iron”, composite PLA as the ferromagnetic filament for the carcinomas (Protopasta, Vancouver, Washington, USA). All organs were printed with 15% gyroid infill, while the stand was printed at 30% gyroid infill for stability. The carcinomas were printed with gyroid infill depending on their size. Further, stainless steel washers (50 mm outer diameter) were glued into a slot at the base of the stand as weights. Different sizes of Neodym magnets (10 mm, 4 mm and 3 mm diameter, all with 2 mm height) were used to hold each carcinoma. A depiction of one 3D printed model is given in Fig. 3.

###  Model validations

At least three specialists from each profession (surgery, pathology, radiology and radiotherapy) with special oncologic experience of over 5 years participated in the study. All participants gave their consent in voluntarily participating in this work. All information gained was anonymized. All groups assessed the five models with an evaluation-form which was individualized for every profession. The questionnaires were divided into three sections. The first section included questions concerning the general analysis of the five models. The second section evaluated the possibilities of application of the 3D printed models in the respective fields (surgery, pathology, radiology and radiotherapy). The third section asked about the physicians’ general experience. Section one and two involved questions with a five-point Likert scale,

dichotomous questions (“Yes” or “No”) and multiple-select questions. Section three only included questions with a five-point rating scale.

### Statistics

Statistical analysis was performed using by SPSS Statistics Version 25.0 (SPSS Inc., Chicago, IL. USA) and Microsoft Excel Version 2023 (Microsoft Corp., Washington, USA).

### Use of large language models

ChatGPT Version 4.0 (OpenAI Inc., San Francisco) was used for language quality check.

## Results

The 3D printed individualized models can be of aid for supporting patient care in case of cervical cancer.

### Common questions for all disciplines

**Fig. 4:**
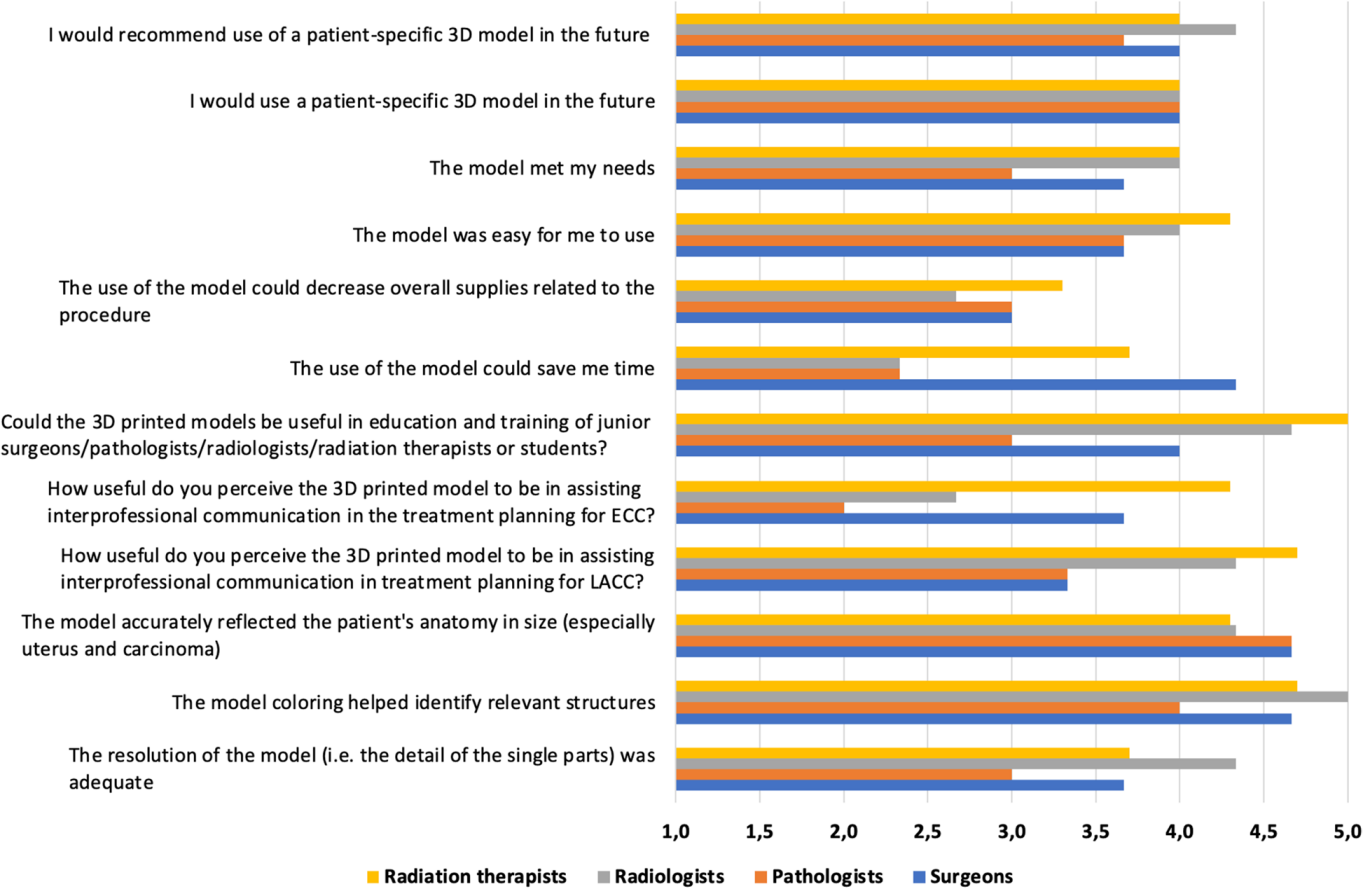
Mean scores of all common questions. 1 = „strongly disagree “ or „not at all useful “ up to 5 = „strongly agree “ or„extremely useful “. Yellow = Radiation therapists, Grey = Radiologists, Orange = Pathologists, Blue = Surgeons

Twelve questions were answered by all disciplines: surgeons, radiologists, pathologists and radiation oncologists (Fig. 4). The first part of the questions was answered by choosing on a scale of one to five (1 = Strongly disagree, 2 = Disagree, 3 = Neither agree, nor disagree, 4 = Agree, 5 = Strongly agree, or 1 = Not at all useful, 2 = Not so useful, 3 = Somewhat useful, 4 = Very useful, 5 = Extremely useful). The three oncologic surgeons, who evaluated the five models, rated the models’ resolution with a mean of 3.7 of 5 points and the helpfulness of the different coloring of the models’ single parts for identifying single structures with a mean of 4.7 of 5 points. The three pathologists also partly agreed to these two aspects (3.0 and 4.0 of 5 points). Radiologists and radiation oncologists rated the detail of the single parts with 4.3 and 3.7 points and the coloring with 5.0 and 4.7 points. The models’ size was assessed as accurate: 4.7 of 5 points from the pathological and surgical point of view and 4.3 of 5 points from the radiological and radiooncological perspective. The surgeons perceived the support generated by the 3D printed models for interprofessional communication in treatment planning for patients with locally advanced cervical cancer (LACC) and early cervical cancer (ECC) as somewhat useful and useful (3.3 and 3.7 of 5 points, respectively). The pathologists rated these aspects with 3.3 and 2.0 points and the radiologists with 4.3 and 2.7 points. The radiation oncologists evaluated the support for interprofessional communication with 4.7 points for LACC and 4.3 points for ECC. The option of utilizing the models for educational purposes for junior colleagues received 4.0 from 5 points from the surgical oncologists, 3.0 points by their pathological colleagues, 4.7 points by radiological specialists and 5.0 points by the radiation oncologists.

Two surgeons agreed and one strongly agreed that the models could save them time, leading to a mean point score of 4.3 of 5 points. Two pathologists disagreed and one neither agreed nor disagreed concerning this matter, making up for 2.3 points. Two radiologists neither agreed nor disagreed and one strongly disagreed, resulting in a mean of 2.3 points. One radiation oncologist neither agreed nor disagreed and two agreed. A mean of 3.7 points was given. All specialties were unsure, if the use of the 3D printed models could decrease overall supplies related to their diagnostic and therapeutical procedures (3.0 of 5 points by surgeons and pathologists, 3.3 of 5 points by radiation therapists and 2.7 points by radiologists). All participants of the study agreed that the models were easy to use, resulting in 3.7 points from the surgery- and pathology-department, 4.0 points from the radiology and 4.3 points from the radiation oncology department. The surgeons answered with mean point score of 3.7 to the question whether the models met their needs. The pathologists answered this question with 3.0 and the radiologists together with the radiation oncologists with a mean of 4.0 points. The surgeons and the radiation oncologists both agreed to using and recommending the use of patient-specific 3D models in the future (mean points of 4.0 each). The pathologists agreed to this in an equal manner (mean 4.0 points for “I would use a patient-specific 3D model in the future” and 3.7 points for “I would recommend use of a patient-specific 3D model in the future”). The radiologists also gave a mean of 4.0 points for using and 4.3 points for recommending the use of patient-specific 3D models in the future.

### Department-specific questions

**Fig. 5:**
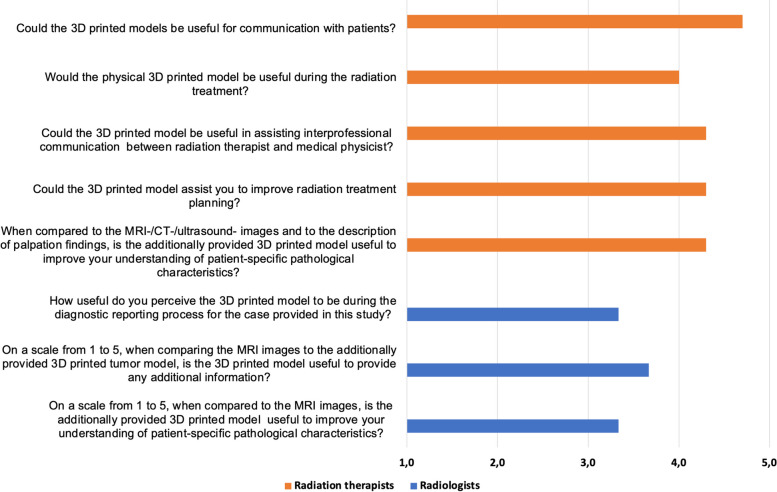
Mean scores of speciality-specific questions. 1 = „strongly disagree “ or „not at all useful “ up to 5 = „strongly agree “ or„extremely useful “. Orange = Radiation therapists, Blue = Radiologist

**Fig. 6:**
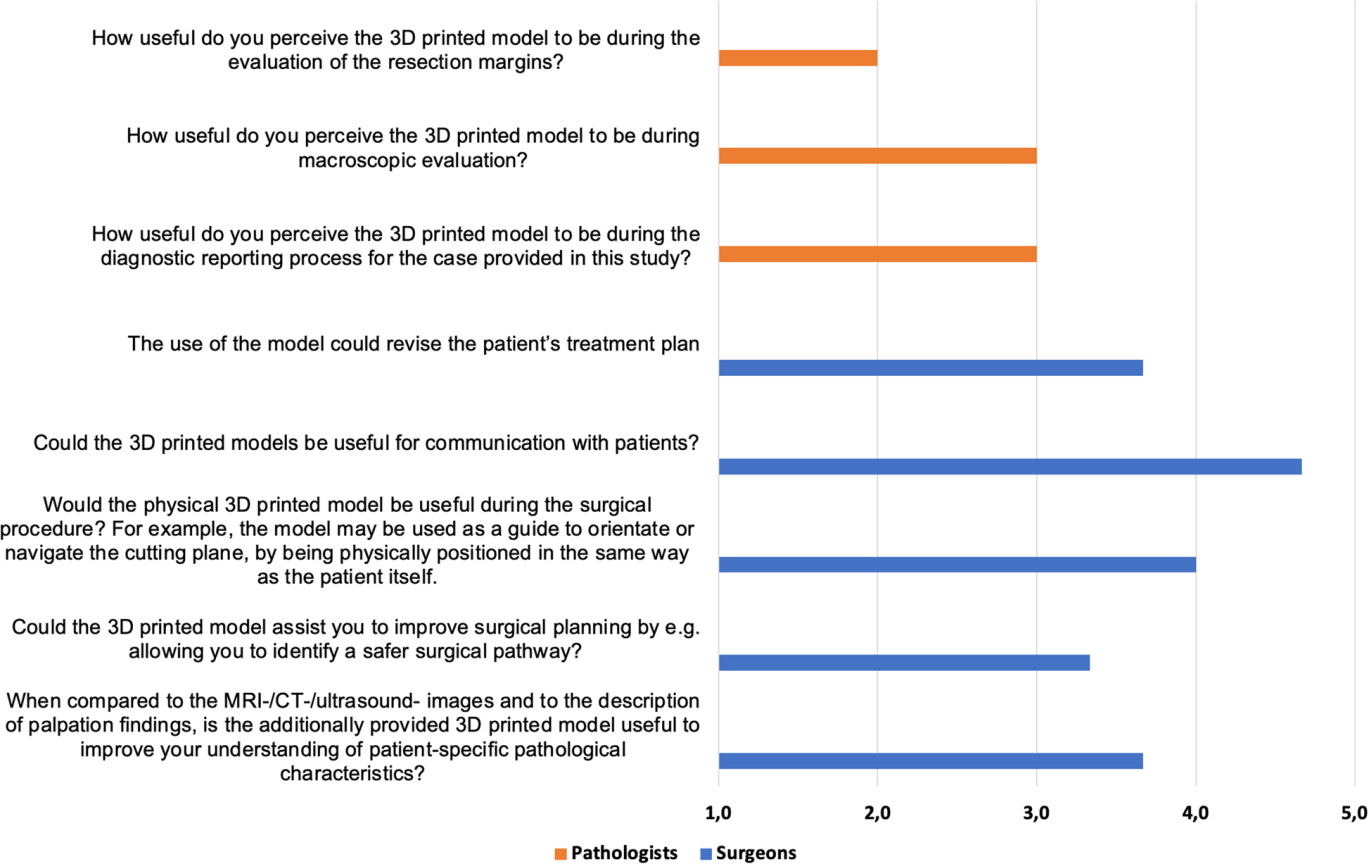
Mean scores of speciality-specific questions. 1 = „strongly disagree “ or „not at all useful “ up to 5 = „strongly agree “ or„extremely useful “. Orange = Pathologists, Blue = Surgeons

Every discipline also answered questions tailored to their everyday clinical work with one part of the questions to be answered by using the rating scale from one to five points as stated above (Figs. 5 and 6). The second part of the questions was answered by using “Yes “ or “No “ statements.

### Department-specific questions: surgeons

The oncologic surgeons rated the question “When compared to the MRI-/CT-/ultrasound-images and to the description of palpation findings, is the additionally provided 3D printed model useful to improve your understanding of patient-specific pathological characteristics?” with a mean point score of 3.7 of 5. The question whether the 3D printed models could assist the surgeon to improve surgical planning by e.g. allowing the identification of a safer surgical pathway received a rating of 3.3 points. The question “Would the physical 3D printed model be useful during the surgical procedure? For example, the model may be used as a guide to orientate or navigate the cutting plane, by being physically positioned in the same way as the patient itself.” scored a mean of 4.0 of 5 points. Two surgeons strongly agreed and one agreed that the models could be useful for communication with patients (4.7 of 5 points). The surgeons rated the question “The use of the model could revise the patient’s treatment plan” with a mean of 3.7 of 5 points. All surgeons agreed that the 3D printed models provided additional information compared to the MRI-/CT-/ultrasound-images and to the description of palpation findings. Moreover, all confirmed, that the models gave a better perception of the depth of local invasion (three times “Yes”). The models also gave a better perception of the spatial relationships between surrounding structures (e.g. bladder, rectum), with all surgeons answering this question with “Yes”. In addition, all surgeons were of the opinion, that understanding the depth of local invasion and the spatial relationship between surrounding structures is important for surgical planning for cervical cancer. The question “Do you believe that 3D printed cervical models could have the potential to reduce the chance of intraoperative complications?” was answered with “Yes” twice and with “Yes, in complex cases only”. Furthermore, every questioned surgeon believed 3D printed cervical models could reduce the duration of surgery.

### Department-specific questions: pathologists

The questioned pathologists rated the question “How useful do you perceive the 3D printed model to be during the diagnostic reporting process for the case provided in this study?” with a mean of 3.0 of 5 points. The same score was given to the question whether the 3D printed models were useful during the macroscopic evaluation of the specimen. The question “How useful do you perceive the 3D printed model to be during the evaluation of the resection margins?” scored 2.0 points. All pathologists believed the models served as a useful supplementary tool in the diagnostic reporting process for patients with cervical cancer in some cases (less than 50%).

### Department-specific questions: radiologists

The department of radiology rated the question “When compared to the MRI-/CT-/ultrasound- images and to the description of palpation findings, is the additionally provided 3D printed model useful to improve your understanding of patient-specific pathological characteristics?” with a mean point score of 3.3 of 5. The question “When comparing the MRI images to the additionally provided 3D printed tumor model, is the 3D printed model useful to provide any additional information?” scored a mean of 3.7 points. The radiologists rated the question of how useful they perceive the 3D printed model to be during the diagnostic reporting process with a mean point score of 3.3. Two radiologists answered the question “When comparing the MRI-/CT-/ultrasound-images to the 3D printed cervix model, does the 3D printed model give you a better perception of the depth of local invasion?” with “Yes” and one radiologist with “They provide the same information”. Two radiologists agreed that the 3D printed cervix model gave a better perception of the spatial relationships between surrounding structures (e.g. bladder, rectum) then MRI-/CT-/ultrasound-images. One radiologist was of the opinion, that they provided the same information. All radiologists stated that understanding the depth of invasion and the spatial relationship between surrounding structures is important in their diagnostic reporting process for cervical cancer. The question “Do you perceive the 3D printed model to be a useful supplementary tool in the diagnostic reporting process for patients with cervical cancer?” was answered once with “Yes, in some cases (less than 50%), once with “No” and once with “Yes, in most cases (more than 50%)”.

### Department-specific questions: radiation oncologists

The physicians of the department for radiation oncologists rated the question “When compared to the MRI-/CT-/ultrasound- images and to the description of palpation findings, is the additionally provided 3D printed model useful to improve your understanding of patient-specific pathological characteristics?” with a mean of 4.3 points of 5 points. The same score was reached by the question “Could the 3D printed model be useful in assisting interprofessional communication between radiation oncologists and medical physicist?” and by the question „Could the 3D printed model assist you to improve radiotherapy treatment planning?”. The question „Could the 3D printed models be useful for communication with patients?” scored a mean of 4.7. The radiation oncologists rated the question of how useful they perceive the 3D printed model to be during radiotherapy treatment with a mean point score of 4.0. All radiation oncologists agreed that the 3D printed model provided additional information compared to the MRI-/CT-/ultrasound-images and to the description of palpation findings. The question “When comparing the MRI-/CT-/ultrasound-images to the 3D printed cervix model, does the 3D printed model give you a better perception of the depth of local invasion?” was answered twice with “No” and once with “They provide the same information”. All radiation oncologists agreed that the 3D printed cervix model, gave a better perception of the spatial relationships between surrounding structures (e.g. bladder, rectum) compared to MRI-/CT-/ultrasound-images. The same confirmation was found for the question “Is understanding the depth of local invasion and the spatial relationship between surrounding structures important for surgical planning for cervical cancer?”. The question “Do you believe that 3D printed cervical models could have the potential to reduce the chance of complications?” was answered with “Yes, the enhanced perception that the printed models provide could further reduce the chance of complications during radiation” twice and once with “Yes, in all cases (complex and relatively simple cases)”.

## Discussion

This work focuses on the application of 3D printed models in the clinical field, particularly emphasizing the use of individualized 3D printed models for the diagnostic process and treatment planning of cervical cancer by surgeons, pathologists, radiologists and radiation oncologists.

### Evaluation of the 3D printed models by common questions

#### Accurate anatomical representation

An important condition for further integration of 3D printed models into clinical practice is an accurate and easily comprehensible representation of the patients’ anatomy. All participants including surgeons, radiologists, pathologists and radiation oncologists evaluated the model resolution (mean scores between 3.0 and 4.3 points out of 5) and its usefulness in identifying specific structures positively (mean scores between 4.0 and 5.0 out of 5). Additionally, the models were considered to be of accurate size, with ratings ranging from 4.3 to 4.7 out of 5, and easy to use, with ratings from 3.7 to 4.3 out of 5, depending on the discipline. Therefore, the condition of accurate and comprehensible anatomy representation was met.

#### Interprofessional communication

Another aspect explored in this study was the potential for 3D printed models to enhance translational oncology by supporting interprofessional communication. Interestingly, opinions varied among radiation oncologists, radiologists, surgeons and pathologists. Radiation oncologists and radiologists highly valued the models for facilitating the exchange between medical professions concerning LACC, rating them with 4.7 and 4.3 points out of 5. However, both surgeons and pathologists saw limited usefulness in these models, with mean scores of 3.3 out of 5. The discrepancy may be due to the fact that the two disciplines, surgery and pathology, are less accustomed to working with replicas and models and typically involve hands-on practice. Tailored training and strategies for integrating 3D models into their workflow could potentially bridge this gap.

#### Usefulness during treatment/diagnostic procedure

The potential for utilizing the models during treatment/diagnostic procedure was also evaluated differently by the four specialty fields: Surgeons and radiation oncologists expressed a high interest with mean scores of 4.0 out of 5, whereas pathologists and radiologist were more critical with mean score of 3.0 and 3.3 out of 5. This is not surprising, considering that both disciplines, radiologists and pathologists, are typically not directly involved in treatment planning. The surgeons’ and radiation therapists’ high interest, in contrast, reflects the potential for enhanced treatment precision and reduced side effects.

#### Educational value

Despite the lack of consensus among the four disciplines regarding the benefits during treatment and diagnostic reporting, there was agreement that 3D printed models could be used and recommended in future, particularly for educational purpose. Remarkably, radiation oncologists expressed the highest enthusiasm for educational value, rating it at 5 points. Surgeons, radiologists, and pathologists also recognized the educational benefits of the models rating with mean scores between 3.0 and 4.7 out of 5. This aligns with the fact that 3D printed models are already used in medicine for educational purposes in various ways, as demonstrated by previous studies (Marconi et al., 2017 and Wake et al., 2019).

### Evaluation of the 3D printed models by department specific questions

#### Surgeons

When examining the utility of 3D printed models in the field of oncologic surgery, the findings reveal a generally positive perception among surgeons. They found the 3D printed models very useful during surgical procedure, such as for guiding surgical orientation and navigation (mean score of 4.0 out of 5). This could be attributed to the surgeons’ discovery that the models offered additional information beyond traditional imaging and palpation findings enhancing the perception of the depth of local invasion and spatial relationships between surrounding structures. Surgeons concluded that 3D printed models could reduce the chance of intraoperative complications and that they could reduce the duration of surgery which has implications for improving patient outcome and resource efficiency. Overall, these findings suggest that, in the surgeons’ opinion, 3D printed cervical models hold promise in enhancing their clinical practice.

#### Pathologist

When examining the utility of 3D printed models in the field of pathology the findings reveal a more critical opinion. Pathologist rated the overall usefulness during the macroscopic evaluation and the diagnostic reporting process with a moderate mean score of 3.0 out of 5. These results underline the fact that 3D printing does not facilitate the examination of the resection margin, which is one of the main questions posed to the pathologists (mean score of 2.0 out of 5). Despite these moderate ratings, all pathologists agreed that the 3D printed models—in a limited number of cases—could be useful as supplementary tools in the diagnostic reporting process. Additional research and refinement of the technology, especially concerning the tumor margins, could lead to a more widespread and effective adoption in pathological practice.

#### Radiologists

When examining the utility of 3D printed models in the field of radiology the findings reveal a mixed opinion. The 3D printed models were considered only moderately useful for improving the understanding of patient-specific pathological characteristics and during the diagnostic reporting process with mean scores of 3.3 out of 5. This is rather surprising as most radiologists agreed that the 3D printed model enhanced their understanding of the spatial relationships between surrounding structures. Moreover, when compared to MRI images the 3D printed tumor model was generally seen as providing additional information, receiving a mean score of 3.7 points out of 5. When asked about the overall usefulness as a supplementary tool, responses were mixed with one radiologist agreeing in some cases, one disagreeing and one agreeing in most cases. This underlines once more that further investigation is needed to optimize the 3D printed models’ role for radiologists.

#### Radiation oncologists

Among radiation oncologists the assessment of 3D printed models for cervical cancer demonstrates highly positive findings. Experts in radiotherapy rated the 3D printed models with 4.3 out of 5 when compared to traditional imaging and palpation findings. Regarding treatment planning and radiotherapy treatment the models received a strong score of 4.3 and 4.0 out of 5, suggesting they are beneficial in the phase of treatment process. This might be due to the fact that the 3D printed models were seen as particularly effective in improving the perception of spatial relationships between surrounding structures and reducing the chance of complications. These findings strongly underline the value of 3D printed models in radiotherapy for cervical cancer. They are perceived as highly effective tools for improving understanding, communication and treatment planning.

### Existing data and limitations

The advancement of 3D printing technology has led to an increasing number of publications assessing the use of 3D printing in medicine. Systematic reviews have outlined its advantages in surgery, such as enhanced pre-operative planning, improved operative outcomes and reduced surgical time [5, 11]. Initially focused on specific surgical specialties, recent reviews, including one from 2023, highlight a growing interest in 3D printing in gynecology, particularly in gynecologic oncology [12–14]. Cooke et al. searched medical databases and systematically reported on the clinical applications of individualized 3D printing in gynecology [12]. 63% of the studies investigated printed five or less models which is equivalent to the number of 3D printed models in our study. Notably, patient specific 3D printed brachytherapy devices emerged as a widely studied application [12, 15]. Studies reported increased radiation doses to the target volume and decreased dose to organs at risk, emphasizing the potential benefits of individualized 3D printing in improving treatment outcomes [15, 16]. This high interest in 3D printing by radiation oncologists is also confirmed by our study results: Physicians in the field of radiooncology rated our 3D printed models with the highest possible score of 5.0 out of 5 when compared to traditional imaging and palpation findings. Regarding treatment planning and radiotherapy, the models received a strong score of 4.0 out of 5, suggesting they are beneficial in the phase of treatment process.

Even though 3D printed models in gynecologic oncology have mainly been utilized for production of patient specific 3D printed brachytherapy guides/ applicators, studies indicate its additional potential in surgical planning and education [6, 12, 17, 18]. To validate this concept initially, Ajao et al. and Mackey et al. generated highly accurate individualized models of endometriotic nodules or fibroids by applying additive manufacturing. These models closely aligned with surrounding tissue and.

and adequately depicted patient anatomy [19, 20]. Studies by Baek et al. and Sayed Aluwee et al. further substantiated this approach, revealing that gynecologic oncologists experienced enhanced comprehension of patient anatomy and pathology, including factors such as tumor size, shape, and borders. The use of individualized 3D printed models notably increased their confidence in determining the optimal route of excision, particularly in the preparatory phase of oncologic surgeries [2, 6]. Overall, these studies highlight the accuracy of 3D printed models in representing gynecologic pathology, aiding surgical comprehension, and increasing confidence in surgical routes. This observation was confirmed by our study results: Surgeons rated the possibility of additional information with a mean point score of 3.7 of 5 and the usefulness during surgical procedure with a mean of 4.0 of 5 points.

In contrast to existing studies, our patient-specific 3D printed models of cervical cancer were evaluated by all medical specialties involved in diagnosis and treatment planning. Thereby we could highlight the differences of opinions and identify the topics which surgeons, radiologists, radiation therapist and pathologists agreed on. Interestingly the benefit for interprofessional communication for instance was rated differently: Radiation oncologists and radiologist highly valued the models for facilitating the exchange between medical professions. Surgeons and pathologist were more reserved. Nevertheless, all specialties agreed that 3D printed models could be used and recommended in future, particularly for educational purpose.

Given the pivotal role of interprofessional collaboration in oncology, we recommend further studies integrating all specialties to comprehensively assess the impact of 3D printing in medical practice.

## Conclusion

All specialty fields agreed on the usefulness of the individualized 3D printed models as supplementary tools. There were mixed responses concerning the utilization for diagnostic processes and treatment planning of cervical cancer by surgeons, pathologists, radiologists and radiation oncologists. As imaging techniques and data processing, particularly supported by artificial intelligence, continue to advance, the integration of 3D printing in medicine is likely to become increasingly prevalent in future. Novel frameworks, which automate the digitization of patient anatomies, facilitate precise and swift three-dimensional reconstructions while ensuring a faithful representation of their physical characteristics. The ability to create accurate, patient-specific models holds considerable potential for personalized medicine, further extending the quality of clinical diagnostics und treatment planning. Furthermore, 3D printed models can be produced at low costs by anyone with access to a 3D printer, which makes it a cost-effective tool for every clinic. The nearly worldwide and quick accessibility combined with minimal resource demands facilitates a more efficient dissemination of gynecologic knowledge and skills among health care providers worldwide. This underlines the importance of this field of research and shows that further investigation should be conducted. Addressing the concerns and tailoring the use of additive manufacturing to the specific needs of each medical specialty will be essential for realizing its full potential in clinical practice**.**

### Supplementary Information

Supplementary Material 1.

## Data Availability

Data is provided within the manuscript or supplementary information files. The datasets used and analysed during the current study are available from the corresponding author on reasonable request.

## Appendix

In the following, the questions are stated, which were used to evaluate the 3D-printed model.

## QUESTIONNAIRE 1 (Radiology)

☐ I have received information regarding this research and had an opportunity to ask questions. I believe I understand the purpose, extent and possible risks of my involvement in this project and I voluntarily consent to take part.

SECTION ONE – Applications of the 3D printed model.

(1) The quality of the model was adequate.

1 = Strongly disagree. 2 = Disagree. 3 = Neither agree nor disagree. 4 = Agree. 5 = Strongly agree.

(2) The resolution of the model was adequate.

1 = Strongly disagree. 2 = Disagree. 3 = Neither agree nor disagree. 4 = Agree. 5 = Strongly agree.

(3) The model coloring helped identify relevant structures.

1 = Strongly disagree. 2 = Disagree. 3 = Neither agree nor disagree. 4 = Agree. 5 = Strongly agree.

(4) The model accurately reflected the patient’s anatomy in size.

1 = Strongly disagree. 2 = Disagree. 3 = Neither agree nor disagree. 4 = Agree. 5 = Strongly agree.

(5) On a scale from 1 to 5, when compared to the MRI images, is the additionally provided

3D printed model useful to improve your understanding of patient-specific pathological

characteristics?

1=Not at all useful. 2=Not so useful. 3=Somewhat useful. 4=Very useful. 5=Extremely

useful.

(6) On a scale from 1 to 5, when comparing the MRI images to the additionally provided

3D printed tumor model, is the the 3D printed model useful to provide any additional

information?

1=Not at all useful. 2=Not so useful. 3=Somewhat useful. 4=Very useful. 5=Extremely

useful.

(7) When comparing the MRI images to the 3D printed cervix model...

(a) Does the 3D printed model give you a better perception of the depth of invasion?

- ☐ YES
- ☐ NO
- ☐ They provide the same information

(b) Does the 3D printed model give you a better perception of the spatial relationship

between surrounding structures (e.g. bladder, rectum)?

- ☐ YES
- ☐ NO
- ☐ They provide the same information

(c) Is understanding the depth of invasion and the spatial relationship between surrounding structures important in your diagnostic reporting process for cervical cancer?

- ☐ YES, information related to both depth and spatial relationships is important.
- ☐ YES, only the depth of invasion.
- ☐ YES, only the spatial relationships between structures.
- ☐ NO, neither is important in surgical planning.

SECTION TWO – Applications of the 3D printed model

(8) How useful do you perceive the 3D printed model to be during the diagnostic reporting

process for the case provided in this study?

1=Not at all useful. 2=Not so useful. 3=Somewhat useful. 4=Very useful. 5=Extremely

useful.

(9) Do you perceive the 3D printed model to be a useful supplementary tool in the

diagnostic reporting process for patients with cervical cancer?

- ☐ YES, in most cases (more than 50%)
- ☐ YES, in some cases (less than 50%)
- ☐ YES, in simple cases
- ☐ YES, in complex cases
- ☐ NO

(10) How useful do you perceive the 3D printed model to be in assisting interprofessional

communication between radiologists and associated health professionals involved in the

treatment planning for patients with locally advanced cervical cancer?

1=Not at all useful. 2=Not so useful. 3=Somewhat useful. 4=Very useful. 5=Extremely

useful.

(11) How useful do you perceive the 3D printed model to be in assisting interprofessional

communication between radiologists and associated health professionals involved in the

treatment planning for patients with early cervical cancer?

1=Not at all useful. 2=Not so useful. 3=Somewhat useful. 4=Very useful. 5=Extremely

useful.

(12) Could the 3D printed models be useful in education and training of junior radiologists or students?

1 = Not at all useful. 2 = Not so useful. 3 = Somewhat useful. 4 = Very useful. 5 = Extremely useful.

(13) Use of the model could save me time.

1 = Strongly disagree. 2 = Disagree. 3 = Neither agree nor disagree. 4 = Agree. 5 = Strongly agree.

(14) Use of the model could decrease overall supplies related to the procedure.

1 = Strongly disagree. 2 = Disagree. 3 = Neither agree nor disagree. 4 = Agree. 5 = Strongly agree.

SECTION THREE—General Experience.

(15) The model was easy for me to use.

1 = Strongly disagree. 2 = Disagree. 3 = Neither agree nor disagree. 4 = Agree. 5 = Strongly agree.

(16) The model met my needs.

1 = Strongly disagree. 2 = Disagree. 3 = Neither agree nor disagree. 4 = Agree. 5 = Strongly agree.

(17) I would use a patient-specific 3D model in the future.

1 = Strongly disagree. 2 = Disagree. 3 = Neither agree nor disagree. 4 = Agree. 5 = Strongly agree.

(18) I would recommend use of a patient-specific 3D model in the future.

1 = Strongly disagree. 2 = Disagree. 3 = Neither agree nor disagree. 4 = Agree. 5 = Strongly agree.

End of questionnaire. Thank you.

## QUESTIONNAIRE 2 (Surgeons)

☐ I have received information regarding this research and had an opportunity to ask questions. I believe I understand the purpose, extent and possible risks of my involvement in this project and I voluntarily consent to take part.

SECTION ONE – Analysis of the 3D printed model.

(1) The resolution of the model (i.e. the detail of the single parts) was adequate.

1 = Strongly disagree. 2 = Disagree. 3 = Neither agree nor disagree. 4 = Agree. 5 = Strongly agree.

(2) The model coloring helped identify relevant structures.

1 = Strongly disagree. 2 = Disagree. 3 = Neither agree nor disagree. 4 = Agree. 5 = Strongly agree.

(3) The model accurately reflected the patient’s anatomy in size.

1 = Strongly disagree. 2 = Disagree. 3 = Neither agree nor disagree. 4 = Agree. 5 = Strongly agree.

(4) When compared to the MRI-/ CT- /ultrasound images and to the description of palpation findings, is the additionally provided 3D printed model useful to improve your understanding of patient-specific pathological characteristics?

1 = Not at all useful. 2 = Not so useful. 3 = Somewhat useful. 4 = Very useful. 5 = Extremely useful.

(5) When compared to the MRI-/ CT- /ultrasound images and to the description of palpation findings, does the 3D printed model provide any additional information?

- ☐ YES
- ☐ NO ☐☐

(6) When comparing the MRI images to the 3D printed cervix model...

(a) Does the 3D printed model give you a better perception of the depth of invasion?

- ☐ YES
- ☐ NO
- ☐ They provide the same information

(b) Does the 3D printed model give you a better perception of the spatial relationships

between surrounding structures (e.g. bladder, rectum)?

- ☐ YES
- ☐ NO
- ☐ They provide the same information

(c) Is understanding the depth of invasion and the spatial relationship between surrounding structures important in surgical planning for cervical cancer?

- ☐ YES, information related to both depth and spatial relationships is important.
- ☐ YES, only the depth of invasion.
- ☐ YES, only the spatial relationships between structures.
- ☐ NO, neither is important in surgical planning.

SECTION TWO – Applications of the 3D printed model.

(7) Could the 3D printed model assist you to improve surgical planning by allowing you to

identify a safer surgical pathway?

1=Not at all useful. 2=Not so useful. 3=Somewhat useful. 4=Very useful. 5=Extremely

useful.

(8) Would the physical 3D printed model be useful during the surgical procedure? For

example, the model may be used as a guide to orientate or navigate the cutting plane, by

being physically positioned in the same way as the patient itself.

1=Not at all useful. 2=Not so useful. 3=Somewhat useful. 4=Very useful. 5=Extremely

useful.

(9) Do you believe that 3D printed cervical models could have the potential to reduce the

chance of intraoperative complications? Note: you may tick more than one box to answer

this question

- ☐ YES, in all cases (complex and relatively simple cases)
- ☐ YES, in complex cases only.
- ☐ YES, in simple cases only.
- ☐ YES, the enhanced perception that the printed models provide could further reduce the chance of intraoperative complications.
- ☐ NO, the 3D reconstructed images give enough information to reduce the chance of intraoperative complications, the printed model would give no extra information to further reduce chances.
- ☐ Unsure.

(10) Do you believe that 3D printed cervical models could have the potential to reduce the duration of surgery? Note: you may tick more than one box to answer this question.

- ☐ YES, in all cases (complex and relatively simple cases).
- ☐ YES, in complex cases only.
- ☐ YES, in simple cases only.
- ☐ YES, the enhanced perception that the printed models provide could further reduce the chance of intraoperative complications.
- ☐ NO, the MRI images give enough information to reduce the chance of intraoperative complications, the printed model would give no extra information to further reduce chances.
- ☐ Unsure.

(11) How useful do you perceive the 3D printed model to be in assisting interprofessional

communication between radiologists and associated health professionals involved in the

treatment planning for patients with locally advanced cervical cancer?

1=Not at all useful. 2=Not so useful. 3=Somewhat useful. 4=Very useful. 5=Extremely

useful.

(12) How useful do you perceive the 3D printed model to be in assisting interprofessional

communication between radiologists and associated health professionals involved in the

treatment planning for patients with early cervical cancer?

1=Not at all useful. 2=Not so useful. 3=Somewhat useful. 4=Very useful. 5=Extremely

useful.

(13) Could the 3D printed models be useful in education and training of junior surgeons or students?

1 = Not at all useful. 2 = Not so useful. 3 = Somewhat useful. 4 = Very useful. 5 = Extremely useful.

(14) Could the 3D printed models be useful for communication with patients?

1 = Not at all useful. 2 = Not so useful. 3 = Somewhat useful. 4 = Very useful. 5 = Extremely useful.

(15) Use of the model could save me time.

1 = Strongly disagree. 2 = Disagree. 3 = Neither agree nor disagree. 4 = Agree. 5 = Strongly agree.

(16) Use of the model could decrease overall supplies related to the procedure.

1 = Strongly disagree. 2 = Disagree. 3 = Neither agree nor disagree. 4 = Agree. 5 = Strongly agree.

(17) Use of the model could revise the patient’s treatment plan.

1 = Strongly disagree. 2 = Disagree. 3 = Neither agree nor disagree. 4 = Agree. 5 = Strongly agree.

SECTION THREE—General Experience.

(18) The model was easy for me to use.

1 = Strongly disagree. 2 = Disagree. 3 = Neither agree nor disagree. 4 = Agree. 5 = Strongly agree.

(19) The model met my needs.

1 = Strongly disagree. 2 = Disagree. 3 = Neither agree nor disagree. 4 = Agree. 5 = Strongly agree.

(20) I would use a patient-specific 3D model in the future.

1 = Strongly disagree. 2 = Disagree. 3 = Neither agree nor disagree. 4 = Agree. 5 = Strongly agree.

(21) I would recommend use of a patient-specific 3D model in the future.

1 = Strongly disagree. 2 = Disagree. 3 = Neither agree nor disagree. 4 = Agree. 5 = Strongly agree.

End of questionnaire. Thank you.

## QUESTIONNAIRE 3 (Radiation therapist)

☐ I have received information regarding this research and had an opportunity to ask questions. I believe I understand the purpose, extent and possible risks of my involvement in this project and I voluntarily consent to take part.

SECTION ONE – Applications of the 3D printed model.

(1) The quality of the model was adequate.

1 = Strongly disagree. 2 = Disagree. 3 = Neither agree nor disagree. 4 = Agree. 5 = Strongly agree.

(2) The resolution of the model was adequate.

1 = Strongly disagree. 2 = Disagree. 3 = Neither agree nor disagree. 4 = Agree. 5 = Strongly agree.

(3) The model coloring helped identify relevant structures.

1 = Strongly disagree. 2 = Disagree. 3 = Neither agree nor disagree. 4 = Agree. 5 = Strongly agree.

(4) The model accurately reflected the patient’s anatomy in size.

1 = Strongly disagree. 2 = Disagree. 3 = Neither agree nor disagree. 4 = Agree. 5 = Strongly agree.

(5) When compared to the MRI images, is the additionally provided 3D printed model

useful to improve your understanding of patient-specific pathological characteristics?

1=Not at all useful. 2=Not so useful. 3=Somewhat useful. 4=Very useful. 5=Extremely

useful.

(6) When compared to the MRI images, does the 3D printed model provide any additional

information?

- ☐ YES
- ☐ NO

(7) When comparing the MRI images to the 3D printed cervix model...

(c) Is understanding the depth of invasion and the spatial relationship between surrounding structures important in surgical planning for cervical cancer?

- ☐ YES, information related to both depth and spatial relationships is important.
- ☐ YES, only the depth of invasion.
- ☐ YES, only the spatial relationships between structures.
- ☐ NO, neither is important in surgical planning.

SECTION TWO – Applications of the 3D printed model.

(8) Could the 3D printed model assist you to improve radiation treatment planning?

1=Not at all useful. 2=Not so useful. 3=Somewhat useful. 4=Very useful. 5=Extremely

useful.

(9) Would the physical 3D printed model be useful during the radiation treatment?

1=Not at all useful. 2=Not so useful. 3=Somewhat useful. 4=Very useful. 5=Extremely

useful.

(10) Do you believe that 3D printed cervical models could have the potential to reduce the

chance of complications?

Note: you may tick more than one box to answer this question

- ☐ YES, in all cases (complex and relatively simple cases)
- ☐ YES, in complex cases only.
- ☐ YES, in simple cases only.
- ☐ YES, the enhanced perception that the printed models provide could further reduce the chance of intraoperative complications.
- ☐ NO, the 3D reconstructed images give enough information to reduce the chance of intraoperative complications, the printed model would give no extra information to further reduce chances.
- ☐ Unsure.

(11) How useful do you perceive the 3D printed model to be in assisting interprofessional

communication between radiologists and associated health professionals involved in the

treatment planning for patients with locally advanced cervical cancer?

1=Not at all useful. 2=Not so useful. 3=Somewhat useful. 4=Very useful. 5=Extremely

useful.

(12) How useful do you perceive the 3D printed model to be in assisting interprofessional

communication between radiologists and associated health professionals involved in the

treatment planning for patients with early cervical cancer?

1=Not at all useful. 2=Not so useful. 3=Somewhat useful. 4=Very useful. 5=Extremely

useful.

(13) Could the 3D printed models be useful in education and training of junior radiation therapists or students?

1 = Not at all useful. 2 = Not so useful. 3 = Somewhat useful. 4 = Very useful. 5 = Extremely useful.

(14) Could the 3D printed models be useful for communication with patients?

1 = Not at all useful. 2 = Not so useful. 3 = Somewhat useful. 4 = Very useful. 5 = Extremely useful.

(15) Use of the model could save me time.

1 = Strongly disagree. 2 = Disagree. 3 = Neither agree nor disagree. 4 = Agree. 5 = Strongly agree.

(16) Use of the model could decrease overall supplies related to the procedure.

1 = Strongly disagree. 2 = Disagree. 3 = Neither agree nor disagree. 4 = Agree. 5 = Strongly agree.

(17) Use of the model could revise the patient’s treatment plan.

1 = Strongly disagree. 2 = Disagree. 3 = Neither agree nor disagree. 4 = Agree. 5 = Strongly agree.

SECTION THREE—General Experience.

(18) The model was easy for me to use.

1 = Strongly disagree. 2 = Disagree. 3 = Neither agree nor disagree. 4 = Agree. 5 = Strongly agree.

(19) The model met my needs.

1 = Strongly disagree. 2 = Disagree. 3 = Neither agree nor disagree. 4 = Agree. 5 = Strongly agree.

(20) I would use a patient-specific 3D model in the future.

1 = Strongly disagree. 2 = Disagree. 3 = Neither agree nor disagree. 4 = Agree. 5 = Strongly agree.

(21) I would recommend use of a patient-specific 3D model in the future.

1 = Strongly disagree. 2 = Disagree. 3 = Neither agree nor disagree. 4 = Agree. 5 = Strongly agree.

End of questionnaire. Thank you.

## QUESTIONNAIRE 4 (Pathologists)

☐ I have received information regarding this research and had an opportunity to ask questions. I believe I understand the purpose, extent and possible risks of my involvement in this project and I voluntarily consent to take part.

SECTION ONE – Analysis of the 3D printed model.

(1) The quality of the model was adequate.

1 = Strongly disagree. 2 = Disagree. 3 = Neither agree nor disagree. 4 = Agree. 5 = Strongly agree.

(2) The resolution of the model was adequate.

1 = Strongly disagree. 2 = Disagree. 3 = Neither agree nor disagree. 4 = Agree. 5 = Strongly agree.

(3) The model coloring helped identify relevant structures.

1 = Strongly disagree. 2 = Disagree. 3 = Neither agree nor disagree. 4 = Agree. 5 = Strongly agree.

(4) The model accurately reflected the patient’s anatomy in size.

1 = Strongly disagree. 2 = Disagree. 3 = Neither agree nor disagree. 4 = Agree. 5 = Strongly agree.

(5) When compared to the MRI images, is the additionally provided 3D printed model

useful to improve your understanding of patient-specific pathological characteristics?

1=Not at all useful. 2=Not so useful. 3=Somewhat useful. 4=Very useful. 5=Extremely

useful.

(6) When compared to the MRI images, does the 3D printed model provide any additional

information?

- ☐ YES
- ☐ NO

(7) When comparing the MRI images to the 3D printed cervix model...

(a) Does the 3D printed model give you a better perception of the depth of invasion?

- ☐ YES
- ☐ NO
- ☐ They provide the same information

(b) Does the 3D printed model give you a better perception of the spatial relationships

between surrounding structures (e.g. bladder, rectum)?

- ☐ YES
- ☐NO
- ☐ They provide the same information

(c) Is understanding the depth of invasion and the spatial relationship between surrounding structures important in surgical planning for cervical cancer?

- ☐ YES, information related to both depth and spatial relationships is important.
- ☐ YES, only the depth of invasion.
- ☐ YES, only the spatial relationships between structures.
- ☐ NO, neither is important in surgical planning.

SECTION TWO – Applications of the 3D printed model.

(8) How useful do you perceive the 3D printed model to be during the diagnostic reporting

process for the case provided in this study?

1=Not at all useful. 2=Not so useful. 3=Somewhat useful. 4=Very useful. 5=Extremely

useful.

(9) Do you perceive the 3D printed model to be a useful supplementary tool in the

diagnostic reporting process for patients with cervical cancer?

- ☐ YES, in most cases (more than 50%)
- ☐ YES, in some cases (less than 50%)
- ☐ YES, in simple cases
- ☐ YES, in complex cases
- ☐ NO

(10) How useful do you perceive the 3D printed model to be in assisting interprofessional

communication between pathologists and associated health professionals involved in the

treatment planning for patients with locally advanced cervical cancer?

1=Not at all useful. 2=Not so useful. 3=Somewhat useful. 4=Very useful. 5=Extremely

useful.

(11) How useful do you perceive the 3D printed model to be in assisting interprofessional

communication between pathologists and associated health professionals involved in the

treatment planning for patients with early cervical cancer?

1=not at all useful 2=not so useful 3=somewhat useful 4=very useful. 5=extremely

useful

(12) Could the 3D printed models be useful in education and training of junior pathologists or students?

1 = Not at all useful. 2 = Not so useful. 3 = Somewhat useful. 4 = Very useful. 5 = Extremely useful.

(13) Use of the model could save me time.

1 = Strongly disagree. 2 = Disagree. 3 = Neither agree nor disagree. 4 = Agree. 5 = Strongly agree.

(14) Use of the model could decrease overall supplies related to the procedure.

1 = Strongly disagree. 2 = Disagree. 3 = Neither agree nor disagree. 4 = Agree. 5 = Strongly agree.

SECTION THREE—General Experience.

(15) The model was easy for me to use.

1 = Strongly disagree. 2 = Disagree. 3 = Neither agree nor disagree. 4 = Agree. 5 = Strongly agree.

(16) The model met my needs.

1 = Strongly disagree. 2 = Disagree. 3 = Neither agree nor disagree. 4 = Agree. 5 = Strongly agree.

(17) I would use a patient-specific 3D model in the future.

1 = Strongly disagree. 2 = Disagree. 3 = Neither agree nor disagree. 4 = Agree. 5 = Strongly agree.

(18) I would recommend use of a patient-specific 3D model in the future.

1 = Strongly disagree. 2 = Disagree. 3 = Neither agree nor disagree. 4 = Agree. 5 = Strongly agree.

End of questionnaire. Thank you

## Abbreviations

2D: Two dimensional
3D: Three dimensional
MRI: Magnetic resonance imaging
LACC: Locally advanced cervical cancer
ECC: Early cervical cancer
