# Supplementary material for: Evaluating the value of individualized 3D printed models for examination, diagnosis and treatment planning of cervical cancer
Source: 3D Print Med. 2024 Jul 27;10:25. doi: 10.1186/s41205-024-00229-8 (PMC11282658; doi:10.1186/s41205-024-00229-8)
Supplement: Supplementary file 1 — Supplementary Material 1. [file 41205_2024_229_MOESM1_ESM.pptx]

## Slide 1
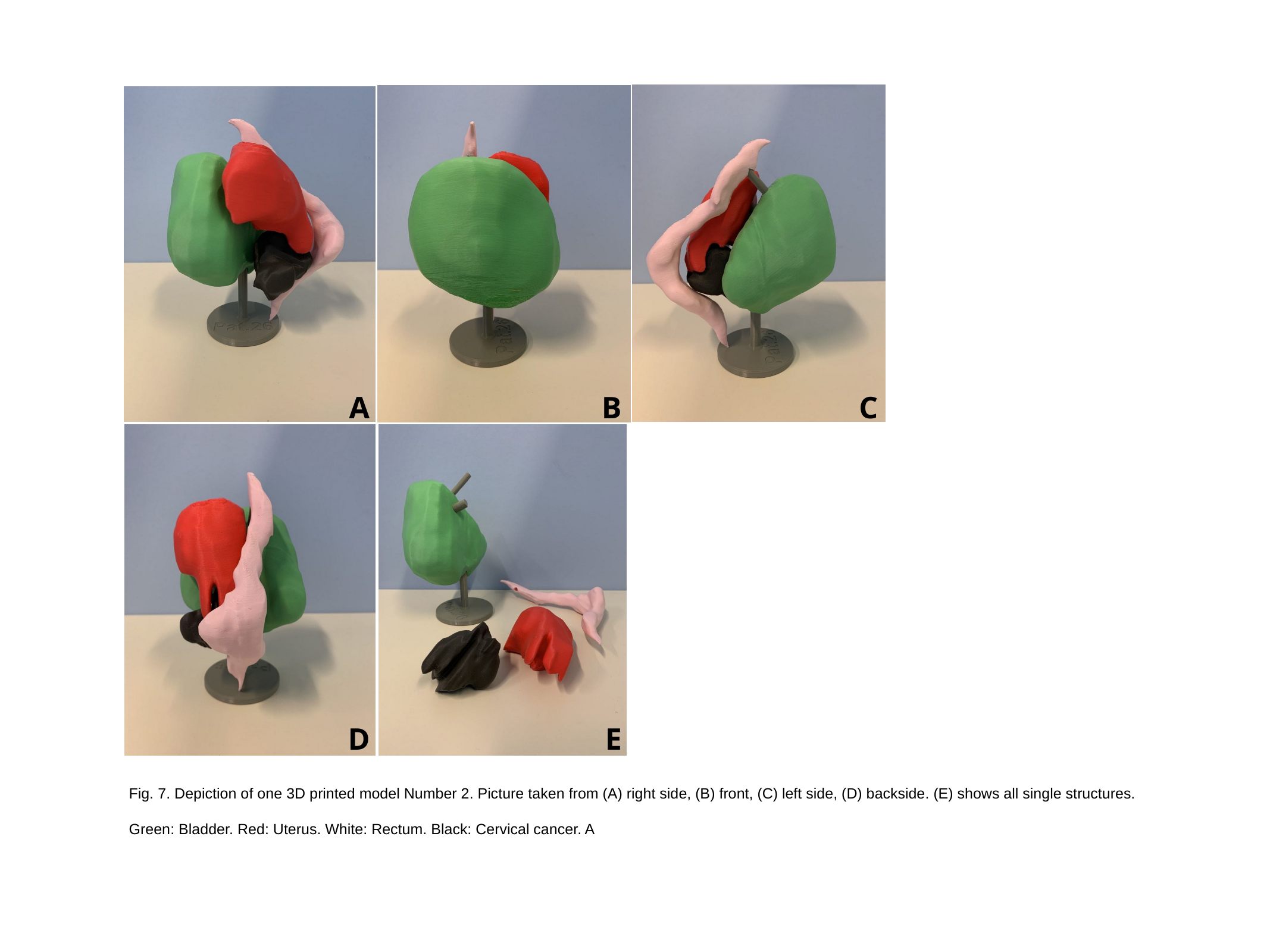

A
B
C
D
E
Fig. 7. Depiction of one 3D printed model Number 2. Picture taken from (A) right side, (B) front, (C) left side, (D) backside. (E) shows all single structures. Green: Bladder. Red: Uterus. White: Rectum. Black: Cervical cancer. A

## Slide 2
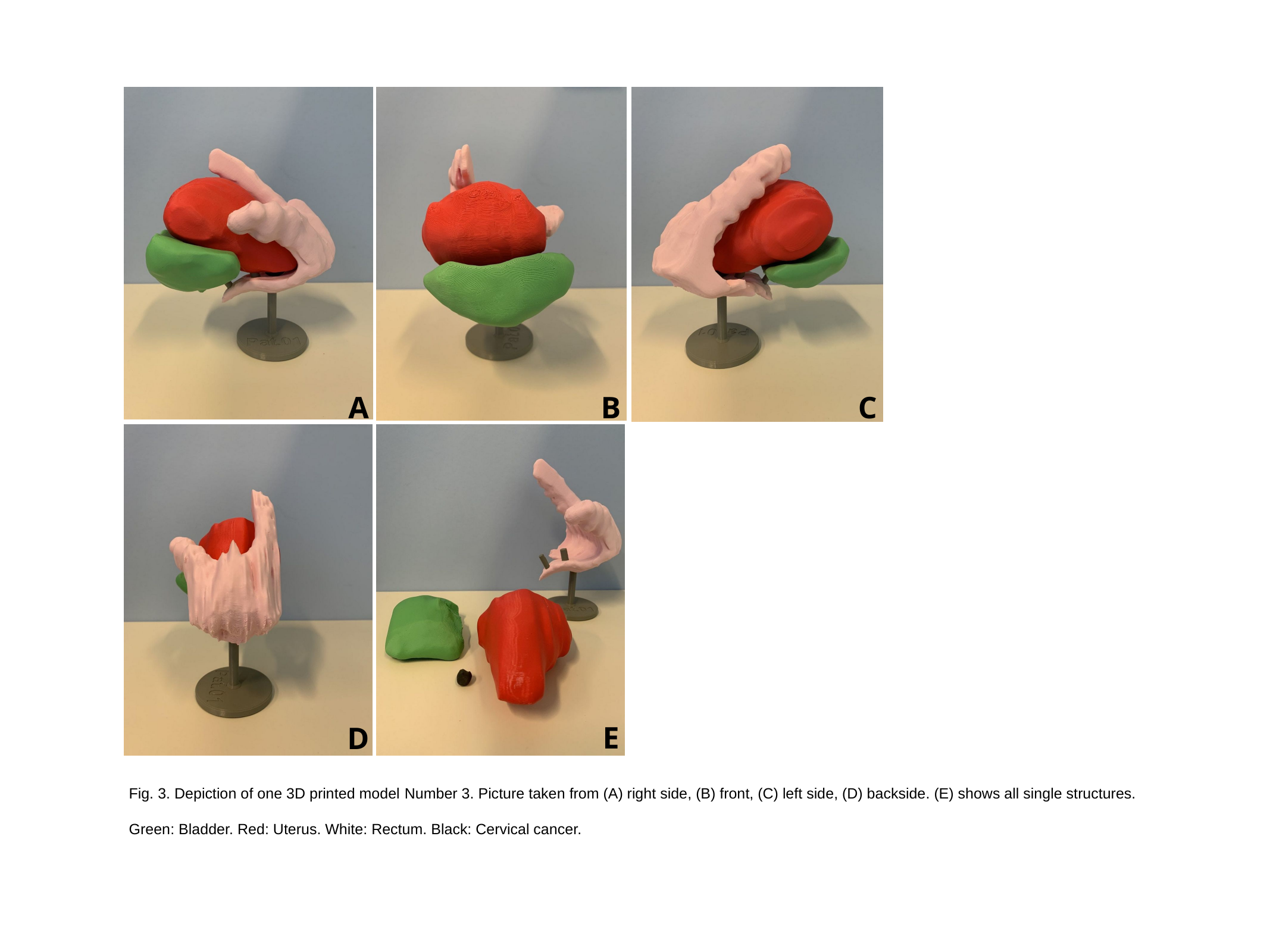

A
B
C
E
D
Fig. 3. Depiction of one 3D printed model Number 3. Picture taken from (A) right side, (B) front, (C) left side, (D) backside. (E) shows all single structures. Green: Bladder. Red: Uterus. White: Rectum. Black: Cervical cancer.

## Slide 3
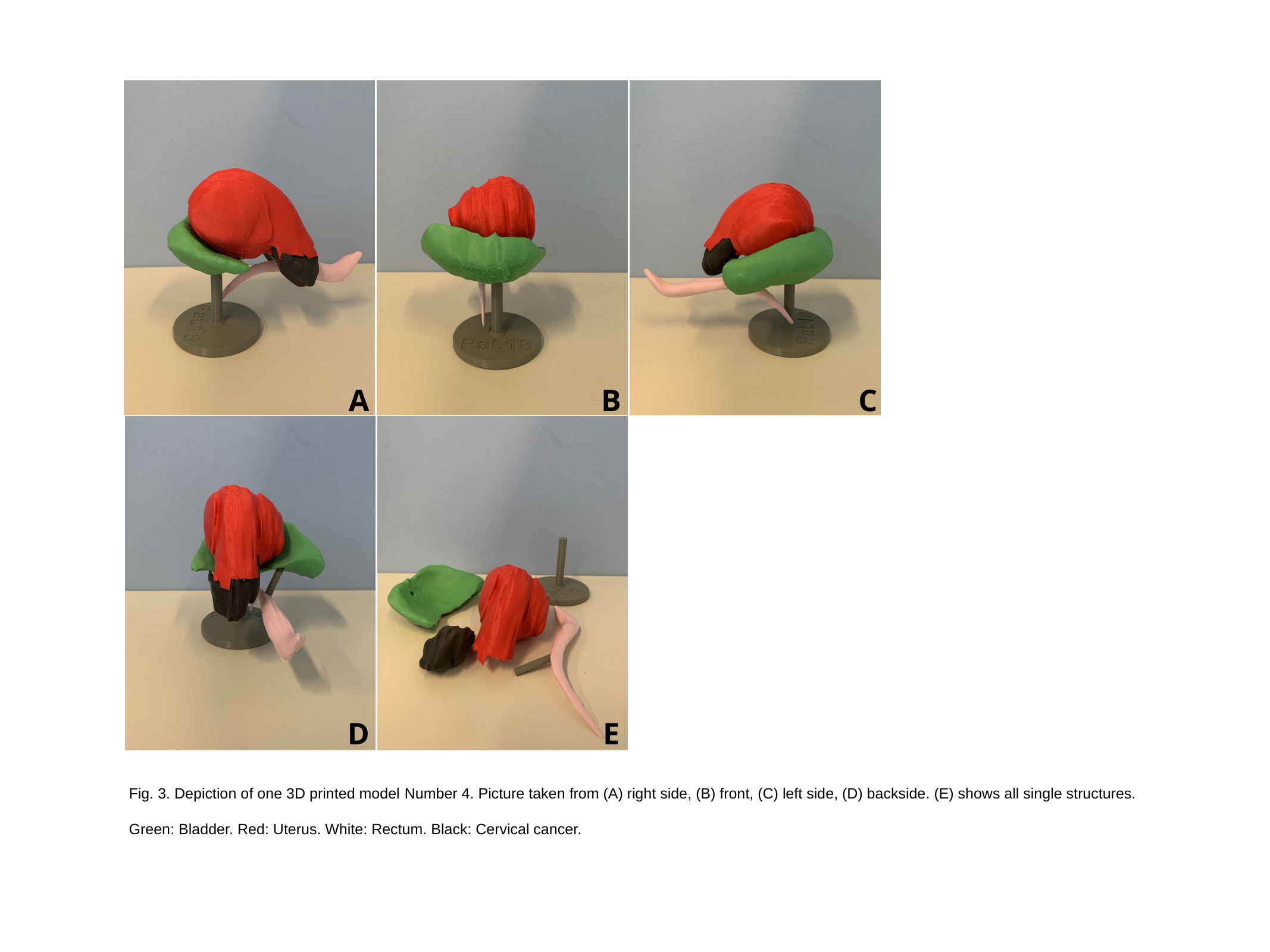

A
B
C
E
D
Fig. 3. Depiction of one 3D printed model Number 4. Picture taken from (A) right side, (B) front, (C) left side, (D) backside. (E) shows all single structures. Green: Bladder. Red: Uterus. White: Rectum. Black: Cervical cancer.

## Slide 4
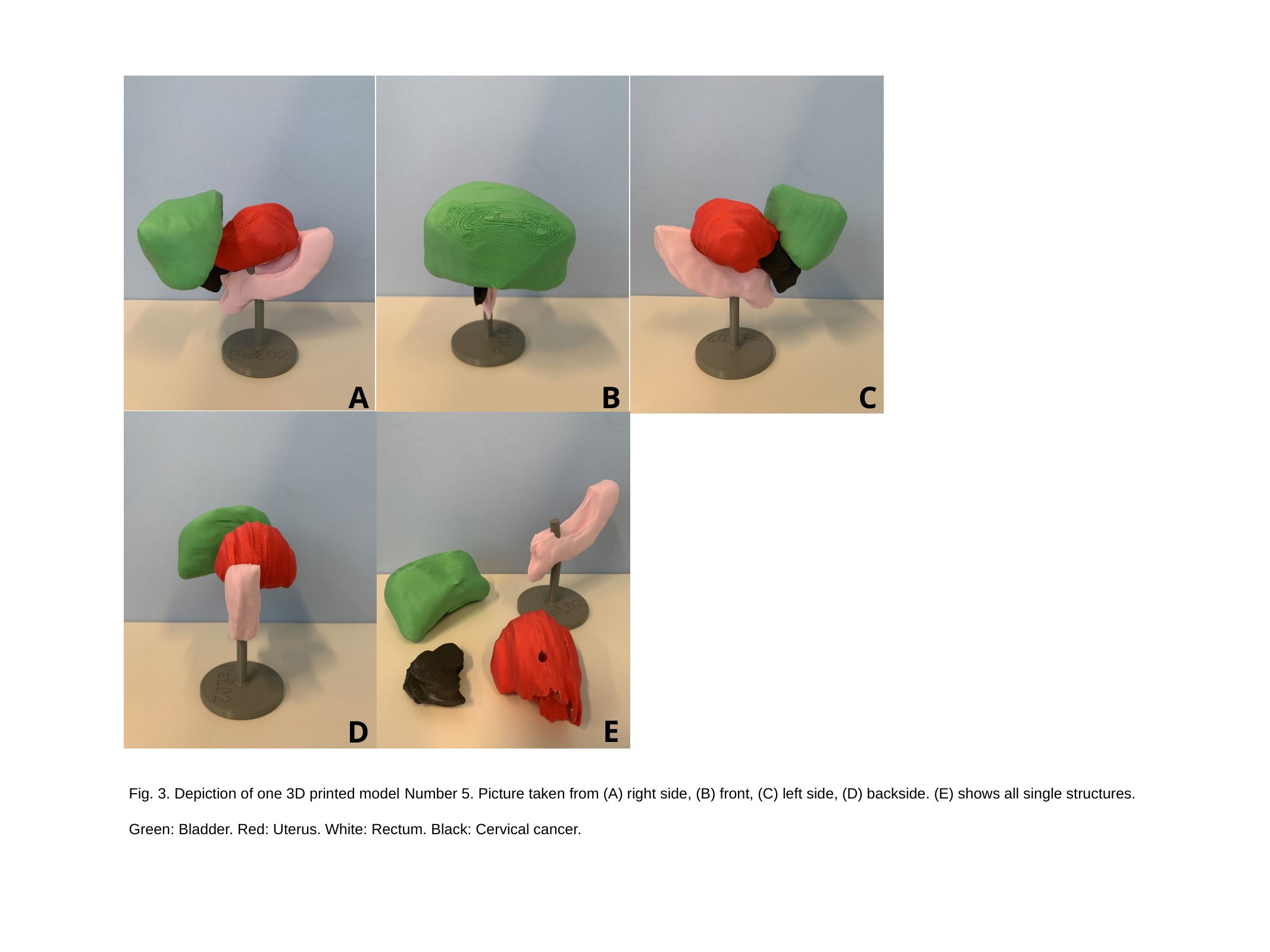

A
B
C
E
D
Fig. 3. Depiction of one 3D printed model Number 5. Picture taken from (A) right side, (B) front, (C) left side, (D) backside. (E) shows all single structures. Green: Bladder. Red: Uterus. White: Rectum. Black: Cervical cancer.
